# Academic achievement of peer group leaders and members: Contributions to adolescents' social, school, and psychological adjustment

**DOI:** 10.1111/jora.70143

**Published:** 2026-01-14

**Authors:** Jiaxi Zhou, Xinyin Chen, Dan Li, Junsheng Liu, Liying Cui, Shihong Liu

**Affiliations:** ^1^ Graduate School of Education University of Pennsylvania Philadelphia Pennsylvania USA; ^2^ School of Psychology Shanghai Normal University Shanghai China; ^3^ School of Psychology and Cognitive Science East China Normal University Shanghai China

**Keywords:** adjustment, Chinese adolescents, nonleader members, peer group leaders

## Abstract

This one‐year longitudinal study examined how the academic achievement of peer group leaders and nonleader members was associated with individual social, school, and psychological adjustment in Chinese adolescents. Participants included 2450 middle‐school students (1233 boys; initial *M*age = 13.96 years) in China. Data were collected from multiple sources, including self‐reports, peer nominations, teacher ratings, and school records. Peer groups and their leaders were identified using the WalkTrap community detection algorithm, resulting in 256 peer group leaders across 238 peer groups. Multilevel modeling revealed that academic achievement of group leaders and nonleader members both positively predicted individual academic achievement and social competence. Whereas group leaders' academic achievement negatively predicted adolescents' aggression and externalizing problems, nonleader members' academic achievement negatively predicted adolescents' peer victimization and internalizing symptoms. The results indicate similar as well as distinct patterns of longitudinal associations of leaders' and nonleader members' academic achievement with individual adjustment in adolescent peer groups. The results suggest that it may be an effective strategy to use peer group‐based education and intervention programs involving group leaders and group members to help adolescents develop social and academic competence and reduce externalizing and internalizing problems.

Adolescence is characterized by an increasing reliance on peers for emotional support and social learning (Hartup, 1989). Most of adolescents' daily interactions occur in self‐selected peer groups formed on common interests and similar attributes (Rubin et al., 2015). Given adolescents' heightened sensitivity to social evaluation and susceptibility to peer influence, peer groups serve as a pivotal socialization context for social, academic, and psychological functioning (Harris, 1995; Kindermann, 2007; Somerville, 2013). However, influence within peer groups is not evenly distributed; group leaders typically occupy central positions, establishing group rules, allocating social resources, and organizing activities (Field et al., 2024; French et al., 2011; Shi & Xie, 2012). Due to their elevated social standing, leaders are presumed to exert a disproportionate impact on their group members' attitudes and behaviors (Gülgöz & Gelman, 2017; Lansford et al., 2009), which is consistent with the status norms perspective (Aguilar‐Pardo et al., 2022). Nonetheless, the majority nonleader group members collectively shape group descriptive norms, representing explicit and implicit standards or expectations that guide group functioning and interactions (Cialdini, 2007; Laninga‐Wijnen et al., 2021). It has been argued that status norms and descriptive norms both play significant roles in individual development, with the former functioning mainly through prestige and visibility and the latter mainly through consensus and conformity (e.g., Aguilar‐Pardo et al., 2022; Cialdini, 2007). Despite the argument about their distinct roles, it is virtually unknown how the attributes of group leaders and nonleader members are associated with adolescents' development.

To fill the gap, the present study sought to examine relations between characteristics of group leaders and nonleader members and adolescents' social, school, and psychological adjustment. We focused on leaders' and nonleader members' academic achievement in this study. Academic achievement is an important indication of students' adaptation to the school environment and an important aspect of peer interaction in early adolescence (Kochenderfer‐Ladd et al., 2022). Research has consistently shown that affiliation with academically high‐achieving peer groups is associated with adolescents' academic motivation, classroom performance, and social competence (Kindermann, 2007; Liu et al., 2023). However, it is unclear how group influence is from group leaders and nonleader group members. In the current one‐year longitudinal study, we examined the differential predictive relations between group leaders' academic achievement and nonleader members' academic achievement and adolescents' subsequent adjustment. By distinguishing the roles of leaders and nonleader members within peer groups and examining their simultaneous associations with individual adjustment outcomes, the study would contribute to the development of a differentiated role framework for the understanding of peer group processes. Moreover, this study would provide valuable information for professionals to design peer‐based educational and intervention programs in schools focusing on different roles of leaders and nonleaders in the group in promoting adolescent adjustment in specific areas (e.g., behavioral, psychological).

## Academic achievement of peer group leaders and nonleader members

In adolescents' informal peer groups, leaders often naturally emerge to organize activities, foster cooperation, navigate challenges, and resolve conflicts (French et al., 2011; Gülgöz & Gelman, 2017; Tackett et al., 2023). Group leaders may exert influence by defining acceptable behaviors and organizing activities in the group (King et al., 2009; Lansford et al., 2009; Shi & Xie, 2012). Group leaders engage in frequent interactions and maintain close relationships with group members and thus may regulate their behaviors more directly and effectively than high‐status students outside the group in the class or school (Zingora et al., 2020). According to the Resource Control Theory (RCT, Hartl et al., 2020), adolescent leadership may be considered a strategic effort aimed at securing valued social and material resources. Adolescents may maintain their leadership status in the group using cooperative strategies (e.g., helping, sharing, and reciprocity), coercive strategies (e.g., aggression and intimidation), or both. Similarly, the prestige‐dominance model indicates that prestige‐oriented leaders may attempt to affect others through displaying their competence and strengths, whereas dominance‐oriented leaders may focus on compliance through threats and fear of consequences (Cheng et al., 2013).

Academic achievement is a salient index of competence among students in middle schools (Chen et al., 2023). Research has demonstrated that academic achievement of adolescents predicts not only their own adjustment but also that of members of their peer groups (Kindermann, 2007; Liu et al., 2023). It has been argued that group members may affect each other on academic achievement through processes such as motivational contagion and collective goal setting (Liu et al., 2023). Group effects on academic achievement may occur in other pathways, including the establishment of achievement‐related group norms, modeling of adaptive behaviors, and provision of academic and emotional support. However, it should be noted that the existing studies did not distinguish different roles of members within peer groups.

Theoretically, it seems reasonable to argue that the academic competence of group leaders reflects their capability to provide academic support and coordinate group learning activities. High‐achieving leaders may motivate members to work hard on academic tasks and provide specific instrumental assistance to them on problem‐solving (Masland & Lease, 2016). Moreover, through organizing academically oriented group activities, group leaders may foster interpersonal skills such as cooperation, communication, help‐seeking behaviors, and conflict resolution among group members (Low & Van Ryzin, 2024). Additionally, leaders who emphasize academic diligence and rule adherence in learning activities likely discourage disruptive and under‐controlled behaviors, whereas nonleader members may lack the authority to directly regulate such behaviors among peers (Gremmen et al., 2019). Leaders also play a central role in organizing group activities both inside and outside the classroom. For example, it has been found that participation in coffeenets or game centers is linked to delinquency and externalizing problems (Xia & Ma, 2023). Academically oriented leaders may steer their groups away from these high‐risk contexts. Relative to group leaders, nonleader members may be less capable of directing group activities or influencing others in the activities. Thus, the academic achievement of group leaders may have cascading effects on adolescents' performance and adjustment in social‐behavioral domains (Masten & Cicchetti, 2010), which is in line with the status norm view that the behaviors and attributes of highly visible and central individuals exert greater influence within the group (Aguilar‐Pardo et al., 2022). Leaders' academic achievement, therefore, functions as a form of prestige that can shape behavioral standards for the group.

Whereas leaders may set group goals and organize group activities, nonleader members in the peer group are likely to represent the norm for regular peer group interactions, such as collaboration and opinion sharing (Cialdini, 2007; Laninga‐Wijnen et al., 2021). The academic performance of nonleader members forms a descriptive norm for how academic success is expected within the group. In groups where most members are academically motivated, adolescents likely adjust their behaviors to fit with this norm, which may foster higher engagement and competence in academic activities. As such, academic achievement of nonleader group members may play a role in creating the achievement‐related group climate, which in turn may be associated with individual social, school, and psychological adjustment. Prior research suggests that an important process involves academic and emotional support among group members (Chen et al., 2023; Liu et al., 2023). Cooperative learning, for example, has been shown to promote collaboration and relatedness, which, at the same time, reduce stress, negative emotions, and social problems such as bullying and victimization (Van Ryzin & Roseth, 2018). Interactions with high‐achieving nonleaders may similarly strengthen motivation and effort to achieve academic success and improve supportive relationships in the group. As the interactions occur on a relatively equalitarian basis, they may be especially effective in helping adolescents develop cooperative social skills and cope with psychological problems (Müller‐Kalthoff et al., 2017). While leaders' achievement may be perceived as a high and extraordinary standard, the experiences and success of nonleader peers may be regarded as more self‐relevant and comparable. According to the social comparison theory, upward comparisons are more likely to enhance self‐evaluations when individuals perceive similarity to better‐performing others, leading to assimilative rather than contrastive responses (Collins, 2000), which in turn promotes adolescents' confidence in performance and adjustment (Jansen et al., 2022).

In short, the literature (e.g., Laninga‐Wijnen et al., 2021; Masland & Lease, 2016) suggests that the academic achievement of peer group leaders may be associated with group functioning and adolescents' development mainly through their roles in organizing group activities, providing directions in the activities, and maintaining standards and expectations for appropriate behaviors. By contrast, the academic achievement of nonleader members may be particularly important for establishing descriptive norms, engaging in social learning, and fostering collaborative interactions and mutual support. Thus, the academic achievement of group leaders and nonleaders may both predict adolescents' social and school competence. At the same time, given their different roles in the group, high‐achieving group leaders may be relatively more inclined to encourage others to control disruptive and other externalizing behaviors, and high‐achieving nonleader peers may be relatively more likely to provide mutual support for adolescents to address adjustment difficulties such as peer victimization and internalizing problems. An examination of associations between leaders and nonleader members' academic achievement and developmental outcomes in different domains would help us move beyond the global group socialization perspective (Harris, 1995) and achieve a more comprehensive understanding of peer group functioning.

## Contributions of group leaders' and members' academic achievement in Chinese adolescents: The present study

Similar to their Western counterparts (e.g., Cairns et al., 1991; Kindermann, 2007), most school‐age children and adolescents in China affiliate with peer groups (Liu et al., 2023). Rooted in Confucian philosophy, Chinese culture places considerable emphasis on collectivist values, including social harmony, interdependence, and respect for authority (Zhao et al., 2024). Peer groups in Chinese schools often exhibit a hierarchical structure (French et al., 2011; Zhou et al., 2024). Group leaders are seen as capable figures who are expected to be more responsible for group organization and activities, and members are expected to work together to achieve common goals.

Academic achievement has been highly valued and emphasized in Chinese society not only as an indicator of personal competence but also as an expression of filial duty and a source of familial honor (Fu et al., 2020). As one of the most important developmental tasks, school‐age children and adolescents in China are required to perform optimally on academic work, and academic success is closely associated with future occupational, economic, and social status. Due to the college entrance examination system, students face strong competition on academic performance as it determines the opportunity to enter senior high schools and colleges or universities. Research has also shown that academic achievement is associated with social and psychological adjustment, such as peer acceptance and perceived self‐worth, among Chinese students (Fu et al., 2020; Yang et al., 2019). The current study examined how academic achievement of peer group leaders and nonleader members would predict adolescents' subsequent academic, social, and psychological adjustment. The emphasis on academic achievement in Chinese schools likely enhances the significance of academic achievement in adolescent peer groups.

Participants in this one‐year longitudinal study were adolescents initially enrolled in grades 7 and 8 in middle schools in rural China. We deliberately selected a rural sample because traditional values (e.g., group orientation) and lifestyles have been more maintained among people in rural regions (approximately 50% of the population in China) than in urban regions (e.g., Liu et al., 2018) and are likely reflected in adolescents' peer group functioning. Middle school in China consists of grades 7 to 9. This developmental period is characterized by increasingly complex peer dynamics, heightened sensitivity to peer evaluation and influence, and escalating academic demands and competitions (Chen et al., 2023; Somerville, 2013). Peer groups and peer group leaders were identified through peer nominations at the initial assessment (Time 1). Adolescents' adjustment in academic, social‐behavioral, and psychological aspects was assessed at both times. As indicated by Kochenderfer‐Ladd and colleagues (e.g., Kochenderfer‐Ladd et al., 2022), students' adjustment is concerned with academic performance, social competence, behavioral control, and psychological well‐being, which are crucial indications of success in the school context.

Based on the literature (e.g., Cialdini, 2007; Laninga‐Wijnen et al., 2021) and the previous discussion, we hypothesized that peer group leaders' academic achievement would positively predict adolescents' subsequent social and school competence and negatively predict later externalizing problems. We also hypothesized that the academic achievement of nonleader group members, as indexed by their average academic scores, would positively predict adolescents' subsequent social and school competence and negatively predict later peer victimization and internalizing problems. To our knowledge, this study is the first to simultaneously examine the longitudinal predictive effects of academic achievement of peer group leaders and nonleader members on adolescent adjustment in multiple domains. We believe that it would help us understand the distinct roles of group leaders and nonleader members in adolescent development.

We checked whether the associations differed by gender or grade. Research has shown that boys may be more likely than girls to conform to dominant or high‐status peers, whereas girls are more relationship‐oriented and susceptible to the influence of group peers (Field et al., 2024; Rose & Rudolph, 2006; Shi & Xie, 2012). Thus, it is possible that the associations between leaders' academic achievement and adjustment outcomes might be stronger in boys than in girls and that the associations between nonleaders' academic achievement and adjustment might be stronger in girls than in boys. Considering the developmental literature indicating that adolescents may become increasingly aware of and sensitive to peer norms (Laursen & Faur, 2022), we expected that the associations of leaders' and nonleaders' achievement with adjustment might be more evident for adolescents in the higher grade.

## METHOD

### Participants

Participants in this study were students, initially in seventh and eighth grades, in five regular public schools that were randomly selected in a region consisting mostly of towns, small cities, and surrounding areas in east China. This region is home to a population of approximately 2.5 million located in the southeast of Anhui province with low mountains and hills. Most families in this region have traditionally lived an agricultural life. Due to urbanization during the economic development and the extension of transport links such as the highway and light rail systems in the past several decades, some residents have engaged in occupations in other sectors such as small business, transportation, and service. The social and economic development has promoted the experiences of the families with more mixed traditional and new values and lifestyles, as in most regions in China (e.g., Chen, 2024).

The initial sample included 2450 students (1233 boys). The mean age of the participants was 13 years and 11 months (SD = 8.30 months). The students were from 48 classes. Public schools primarily serve students in their respective local communities. All schools follow the nationally mandated curriculum and requirements set by China's Ministry of Education. The structure and organization of schools are similar in the country. Students typically stay in the same class throughout middle school. A head teacher who teaches a core subject is assigned to oversee students' academic and daily activities. Students are encouraged to participate in extensive extracurricular activities, creating a variety of opportunities for them to interact with each other.

In the sample, 85.9% of fathers and 92.4% of mothers had a junior high school education or lower and 14.1% of fathers and 7.6% of mothers had a senior high school education or higher. Most participants were of Han ethnicity, which constitutes over 90% of China's population. The demographic data were similar to those reported by the China State Statistics Bureau for populations in similar regions in China (e.g., China State Statistics Bureau, 2014). The demographic variables did not have significant effects on the study variables or their relations.

From the original sample, 2154 adolescents (87.9%) participated in a follow‐up study one year later. A multivariate analysis of variance (MANOVA) revealed no significant differences between adolescents who participated in the follow‐up and those who did not on the Time 1 variables, Wilks' *λ* = .995, *F*(8, 2356) = 1.46, *p* = .166.

### Measures

#### Peer groups and peer group leaders

**FIGURE 1:**
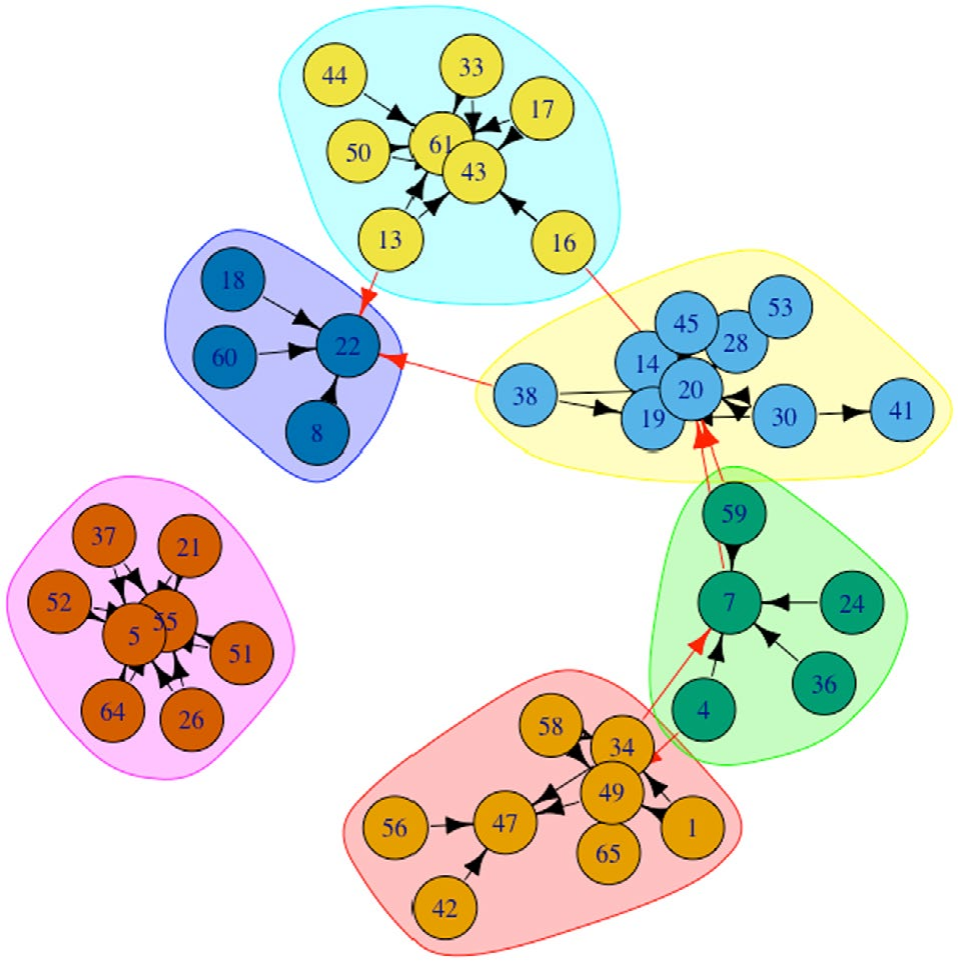
Leadership nomination network in a single classroom, with peer groups identified through the Walktrap clustering algorithm. Each node represents a student, and directed arrows denote leadership nominations. Colored boundaries (and node shading) indicate the distinct peer groups detected by the algorithm.

Peer group and peer group leadership were assessed using the Social‐Cognitive Map (SCM) procedure (Cairns et al., 1991). Participants responded to the following questions: “Do you often hang around with a group in your class? If so, who are they?” and “If there is a leader (or leaders) in the group, who are they?” Participants were allowed to nominate an unlimited number of classmates, regardless of gender, as peer group members or leaders. Participants were instructed to nominate their most important group and had the option to abstain from making nominations. Responses from all participants were used to construct directed and weighted peer group leadership networks. These networks captured both leader–nominator relationships and group member relationships within each identified group. For instance, if Student A nominated Student B as a leader of a group that included Students A, B, C, and D, the network matrix represented not only the connection from A to B, but also connections from C to B and D to B. Following the procedure in previous studies (e.g., Zhou et al., 2024), students who participated but did not nominate others or were not nominated by others (i.e., isolates) were included in the study. Figure 1 illustrates an example of a group leadership network, where nodes represent students and directed edges indicate leadership nominations.

The WalkTrap community detection algorithm (Pons & Latapy, 2005) was applied using the igraph package in R to identify peer groups within the leadership network in each classroom (Csardi & Nepusz, 2006). WalkTrap is a random walk‐based clustering algorithm that detects network communities by analyzing the probability of nodes co‐occurring in short random walks. The algorithm operates under the assumption that random walks tend to remain within the same community as nodes within communities are likely to be connected by shorter random walks, thereby allowing for the identification of structurally cohesive subgroups. Through an iterative process, nodes are merged into communities based on their similarity in random walk patterns, resulting in a hierarchical community structure. Communities are defined at the cut of the dendrogram that maximizes modularity, a widely used index of clustering quality (Smith et al., 2020). The main tuning parameter is the random walk length, which we set to four steps following recommendations in prior work (Pons & Latapy, 2005). As recommended by Letina et al. (2024), WalkTrap is well suited for adolescent peer group research as it captures the traditional notion of peer groups as cohesive and distinct units of interaction. In this approach, the algorithm accounts for directionality in network connections, which is particularly relevant given that leader–nonleader relationships in peer groups are inherently directional (i.e., leadership nominations flow from group members to their leaders). WalkTrap identifies clusters of nodes that are frequently visited together, effectively capturing shared membership within peer groups. For example, if several adolescents are consistently connected through nominations directed toward a specific group leader, both the leader and group members are identified as part of the same group. WalkTrap produces exclusively assigned groups, which align with the hierarchical multilevel modeling (MLM) employed in this study to examine the predictive effects of leaders' characteristics on group members' adjustment. Research on peer networks has demonstrated that WalkTrap produces reliable and valid representations of adolescent peer groups that correspond to observed classroom cliques and friendship groups (e.g., Letina et al., 2024).

Consistent with previous research (e.g., Liu et al., 2023), peer groups consisting of only two members (15 groups; 30 individuals in total) and individuals who were either isolates or members of groups without identifiable leaders (929 individuals) were excluded from the formal analyses. The final analytical sample comprised 1491 individuals in 238 groups. Among them, 82 were all‐boy groups, 93 were all‐girl groups, and 63 were mixed‐gender groups. The average peer group size was 6.26 members, with sizes ranging from 3 to 19 individuals. Figure 1 illustrates group identification based on the group leadership network in a classroom. To identify peer group leaders, we selected the individual within each group who received the highest number of leadership nominations. In cases where multiple individuals received the highest number of nominations in the group, all of them were designated as group leaders of that group. This approach resulted in the identification of 256 group leaders (119 boys and 137 girls).

#### Academic achievement

Data regarding academic achievement in Chinese, mathematics, and English, key subjects in Chinese schools, were obtained from school records. Each subject's maximum score was 100, with 60 typically as the threshold for pass and fail. In the current study, the scores for Chinese, mathematics, and English were significantly correlated (*r* = .54–.70, *p* < .001). Following procedures in previous studies (e.g., Fu et al., 2020), scores for these subjects were standardized within the class. The measure was used and shown to be reliable and valid in previous studies with Chinese children and adolescents (e.g., Chen et al., 2023; Fu et al., 2020; Liu et al., 2023). The internal reliabilities for academic achievement in this study were .83 and .84 at Times 1 and 2, respectively.

#### Peer‐assessed social competence and aggression

Adolescents' social competence and aggression were assessed using a peer assessment measure adapted from *The Revised Class Play* (Masten et al., 1985). The measure included 11 items for social competence (e.g., “Is willing to help others”) and six items for aggression (e.g., “Picks on other kids”). Nominations received from all classmates were used to compute item scores for each student. The item scores were standardized within the class to adjust for differences in the number of nominators. The variables of social competence and aggression were formed based on the corresponding items, with higher scores indicating greater levels of social competence or aggression. The measure was used and shown to be reliable and valid in previous studies with Chinese adolescents (e.g., Zhao et al., 2024; Zhou et al., 2024). Internal reliabilities were .88 and .90 for social competence and .88 and .87 for aggression at Times 1 and 2, respectively, in the present study.

#### Peer victimization

Peer victimization was assessed using a peer nomination measure (Schwartz et al., 2001). Participants were asked to nominate up to three classmates to fit each of four descriptors (e.g., “Gets picked on or teased by other kids”). Nominations received from all classmates were summed to compute the item scores for each participant. The item scores were averaged and standardized within the class to form an index of peer victimization, with higher scores indicating higher peer victimization. The measure was used and shown to be reliable and valid in previous studies with Chinese students (e.g., Liu et al., 2019). The internal reliabilities of this measure were .82 and .80 at Times 1 and 2 in the present study.

#### Teacher‐rated school competence and externalizing and internalizing problems

The head teacher of each class was asked to evaluate each student on school competence, externalizing problems, and internalizing problems using a measure adapted from Hightower et al. (1986). Teachers were asked to rate each student on a 5‐point scale, ranging from 1 (*not at all*) to 5 (*very well*), in terms of how well each item described the student. The measure comprised 13 items for school competence (e.g., “Participates in class discussion”), six items for externalizing problems (e.g., “Disruptive in class”), and seven items for internalizing problems (e.g., “Anxious, worried”). The item scores were standardized within the class to adjust for the teacher's response style and to allow for appropriate comparisons. The average score was computed, with higher scores indicating higher levels of school competence, externalizing problems, or internalizing problems. The measure was used and shown to be reliable and valid in previous studies with Chinese students (e.g., Zhao et al., 2024). The internal reliabilities of the measure in the present study were .85 and .86 for school competence, .77 and .77 for externalizing problems, and .71 and .73 for internalizing problems, at Times 1 and 2, respectively.

#### Depression

Adolescents' depression was assessed using a Chinese version of Kovacs's *Childhood Depression Inventory* (1992). There were 14 items pertaining to a given thought, feeling, or behavior associated with depression (e.g., self‐hate, sadness, self‐blame, fatigue, anhedonia, and reduced appetite). For each item, participants were asked to choose the one that best described them in the past 2 weeks from three alternative responses (e.g., “I feel like crying every day,” “I feel like crying most days,” “I feel like crying once in a while”). Response to each item was scored as 0, 1, and 2, with higher scores indicating greater depression. The average score of the items was computed, with higher scores indicating greater depression. The measure was used and shown to be reliable and valid in previous studies with Chinese children and adolescents (e.g., Chen et al., 2023). The internal reliabilities of this measure were .81 and .82 at Times 1 and 2, respectively, in this study.

### Procedure

Peer group and group leader nominations, peer assessment measures of social competence, aggression, and peer victimization, and a self‐report measure of depression were group administered in the classroom during regular school hours. Teachers completed a rating scale for each student. Academic achievement data were obtained from school records. The study was approved by the Institutional Review Board (IRB). All students in the participating schools were invited to participate without exclusion criteria. Active written consent was obtained from both students and their parents through the school. The participation rate was approximately 95% at each time. The measures were administered by a team of psychology faculty and graduate students in China. Detailed explanations of the procedures were provided during administration. No evidence suggested that students had difficulty understanding the procedures or the items in the measures. Data on peer group and group leader nomination and the adjustment measures were collected at Time 1 in 2013, and data on the same adjustment measures were collected in the follow‐up study one year later.

### Data analytic plan

Multilevel modeling was used mainly to examine the predictive effects of peer group leaders' academic achievement and the average academic achievement of nonleader group members on individual adjustment outcomes. The analysis was conducted separately for each adjustment variable. The analyses were conducted using Mplus 8.3 within a multilevel structural equation modeling (SEM) framework, employing maximum likelihood robust (MLR) estimation to account for nonnormality and model robustness. Each model was specified as a two‐level random‐intercept and random‐slope model, where Level 1 represented individuals, and Level 2 represented peer groups. At Level 1 (individual‐level), leadership status at Time 1 (0 = nonleader, 1 = leader) was included as a predictor, and the corresponding adjustment variable at Time 1 and academic achievement at Time 1 were included as control variables. At Level 2 (group‐level), group leaders' academic achievement and the average academic achievement of nonleader group members were included as predictors and group size, group gender composition, and grade level (0 = Grade 7, 1 = Grade 8) were included as control variables. Group gender composition was determined based on the predominant gender in the group (0 = predominantly male, 1 = predominantly female). To ensure appropriate model specification, individual‐level variables (corresponding adjustment and academic achievement at Time 1) were group‐mean centered, allowing the Level 1 coefficients to be interpreted as the effect of an individual's deviation from their group's mean, and group‐level predictors (leaders' academic achievement, average nonleader members' academic achievement, and group size) were grand‐mean centered, allowing Level 2 effects to be interpreted relative to the overall sample mean. This centering strategy reduces potential confounding between within‐ and between‐group effects and improves interpretability of the results (Enders & Tofighi, 2007). A schematic representation of the conceptual model, including individual‐ and group‐level predictors of adolescents' adjustment, is shown in Figure S1 of the Supplementary Materials. The multilevel model equation examining the effects of individual‐ and peer group‐level variables at Time 1 on individual‐level adjustment at Time 2 is also presented in the Supplementary Materials.

Prior to running the multilevel models, we examined correlations among the group‐level predictors to assess potential overlap. Leaders' and nonleaders' academic achievement were positively but modestly correlated (*r* = .27, *p* < .001), supporting the decision to model them as distinct predictors. Variance inflation factors (VIFs) for models including both predictors were close to 1, suggesting that multicollinearity was not a concern.

## RESULTS

### Descriptive data

The Little's MCAR test (Little, 1988) for the missing data, which ranged from 0% (peer nominations) to 24.3% (academic scores), indicated that the data were missing completely at random, *χ* ^2^(249) = 284.48, *p* = .061. As recommended by Graham (2009), full information maximum likelihood (FIML) estimation was employed to handle missing data. Univariate analyses indicated that girls scored higher on academic achievement, social competence, internalizing problems, and depression and lower on aggression, externalizing problems, and peer victimization than boys at both times. The means and standard deviations for boys and girls are presented in Table 1.

**TABLE 1:** Means and standard deviations of variables.

	Boys		Girls		*F*‐value
	Mean	SD	Mean	SD	
Time 1					
Academic achievement	−0.11	0.91	0.11	0.78	32.26***
Social competence	−0.03	0.65	0.04	0.68	5.73*
School competence	−0.02	0.61	0.02	0.58	1.63
Aggression	0.22	0.96	−0.22	0.36	225.30***
Externalizing problems	0.20	0.72	−0.20	0.56	229.60***
Peer victimization	0.15	0.96	−0.15	0.56	87.44***
Internalizing problems	−0.04	0.59	0.04	0.61	9.49**
Depression	0.38	0.29	0.43	0.30	17.25***
Time 2					
Academic achievement	−0.08	0.91	0.12	0.77	14.65***
Social competence	−0.03	0.65	0.04	0.71	5.60*
School competence	0.00	0.60	0.03	0.60	1.07
Aggression	0.22	1.00	−0.21	0.36	178.40***
Externalizing problems	0.18	0.72	−0.21	0.51	159.50***
Peer victimization	0.12	0.90	−0.12	0.61	51.88***
Internalizing problems	−0.05	0.59	0.03	0.62	6.72**
Depression	0.39	0.30	0.44	0.30	13.70***

* *p* < .05.

** *p* < .01.

*** *p* < .001.

Intraclass correlations (ICCs), which represent the proportion of between‐group (peer group‐level) variance relative to the total variance, ranged from .03 (internalizing problems) to .23 (academic achievement). Preliminary multilevel analyses indicated that, at Time 1, group leaders had higher scores on academic achievement, social competence, school competence, and aggression and lower scores on internalizing problems than nonleader group members. The results are presented in Table S1 of the Supplementary Materials. The correlations among adjustment variables are presented in Table 2. The correlations were weak to moderate, suggesting that the measures assessed distinct but related aspects of adjustment.

**TABLE 2:** Correlations among variables.

	1.	2.	3.	4.	5.	6.	7.	8.	9.	10.	11.	12.	13.	14.	15.	16.
*Time 1*																
1. Academic achievement																
2. Social competence	.43***															
3. School competence	.37***	.36***														
4. Aggression	−.11***	.10***	.02													
5. Externalizing problems	−.25***	−.10***	−.10***	.37***												
6. Peer victimization	−.26***	−.07**	−.12***	.36***	.22***											
7. Internalizing problems	−.05	−.12***	−.16***	−.16***	.10***	.02										
8. Depression	−.16***	−.14***	−.21***	.00	.07**	.12***	.19***									
*Time 2*																
9. Academic achievement	.73***	.39***	.32***	−.07	−.21***	−.25***	−.07	−.19***								
10. Social competence	.40***	.84***	.34***	.07**	−.09***	−.09***	−.09**	−.11***	.38***							
11. School competence	.32***	.34***	.33***	.05	−.08**	−.15***	−.12***	−.22***	.42***	.40***						
12. Aggression	−.15***	.07**	.00	.78***	.34***	.29***	−.14***	.03	−.07	.08**	.02					
13. Externalizing problems	−.18***	−.06	−.06*	.42***	.41***	.22***	−.08*	.04	−.24***	−.05	−.11***	.44***				
14. Peer victimization	−.27***	−.09***	−.11***	.26***	.14***	.69***	−.03	.09***	−.24***	−.11***	−.16***	.32***	.17***			
15. Internalizing problems	−.09**	−.11***	−.11***	−.13***	−.04	.04	.29***	.20***	−.14***	−.10***	−.16***	−.13***	.12***	.01		
16. Depression	−.14***	−.15***	−.17***	.00	.09***	.08**	.18***	.58***	−.20***	−.18***	−.30***	.04	.12***	.09***	.22***	

* *p* < .05.

** *p* < .01.

*** *p* < .001.

### Longitudinal multilevel analyses

The results of the longitudinal multilevel analyses, examining the predictive effects of individual‐ and peer group‐level variables at Time 1 on adjustment variables at Time 2, are presented in Table 3. Findings showed that all adjustment variables had significant stability from Time 1 to Time 2. After accounting for individual‐level Time 1 adjustment, academic achievement, group size, group gender, and grade, Time 1 group leadership status was positively associated with Time 2 social competence and school competence and negatively associated with Time 2 depression. Time 1 group leaders' academic achievement was positively associated with Time 2 academic achievement, social competence, and school competence and negatively associated with Time 2 aggression and externalizing problems. Time 1 nonleader group members' academic achievement was positively associated with Time 2 academic achievement, social competence, school competence, and negatively associated with peer victimization, internalizing problems, and depression.

**TABLE 3:** Predictive effects of individual‐level variables and peer group‐level variables on individual adjustment outcomes.

T2 outcome variable	*B*	SE	*t* value	95% CI
T1 predictor				
Academic achievement				
Individual‐level				
Stability	0.74	0.06	12.83***	(0.62, 0.85)
Leadership status	0.07	0.06	1.12	(−0.05, 0.18)
Group‐level				
Gender	0.06	0.06	1.01	(−0.06, 0.19)
Group size	0.01	0.01	1.18	(−0.01, 0.03)
Grade	0.01	0.08	0.19	(−0.13, 0.16)
Leader's academic achievement	0.16	0.06	2.72**	(0.04, 0.27)
Nonleader's academic achievement	0.50	0.09	5.43***	(0.32, 0.68)
Social competence				
Individual‐level				
Stability	0.08	0.02	3.68***	(0.04, 0.13)
Academic achievement	0.07	0.06	1.15	(−0.05, 0.20)
Leadership status	0.79	0.03	26.43***	(0.73, 0.84)
Group‐level				
Gender	0.01	0.06	0.19	(−0.10, 0.12)
Group size	0.01	0.01	1.79	(0.00, 0.03)
Grade	0.08	0.06	1.26	(−0.04, 0.19)
Leader's academic achievement	0.17	0.04	4.05***	(0.09, 0.25)
Nonleader's academic achievement	0.25	0.06	3.90***	(0.12, 0.37)
School competence				
Individual‐level				
Stability	0.19	0.04	4.34***	(0.10, 0.28)
Academic achievement	0.15	0.07	2.02*	(0.01, 0.30)
Leadership status	0.37	0.05	7.10***	(0.27, 0.48)
Group‐level				
Gender	0.08	0.07	1.18	(−0.06, 0.22)
Group size	0.00	0.01	0.38	(−0.01, 0.02)
Grade	0.05	0.08	0.61	(−0.11, 0.21)
Leader's academic achievement	0.13	0.05	2.49*	(0.03, 0.23)
Nonleader's academic achievement	0.19	0.07	2.69**	(0.05, 0.33)
Aggression				
Individual‐level				
Stability	1.03	0.06	18.09***	(0.92, 1.14)
Academic achievement	−0.07	0.03	−2.83**	(−0.12, −0.02)
Leadership status	0.09	0.06	1.50	(−0.03, 0.21)
Group‐level				
Gender	−0.36	0.07	−5.45***	(−0.50, −0.23)
Group size	0.01	0.01	1.52	(0.00, 0.03)
Grade	−0.06	0.07	−0.90	(−0.20, 0.07)
Leader's academic achievement	−0.10	0.05	−1.98*	(−0.20, −0.01)
Nonleader's academic achievement	−0.02	0.07	−0.32	(−0.16, 0.11)
Externalizing problems				
Individual‐level				
Stability	0.30	0.04	6.86***	(0.22, 0.39)
Academic achievement	−0.07	0.03	−2.21*	(−0.13, −0.01)
Leadership status	0.02	0.06	0.29	(−0.10, 0.13)
Group‐level				
Gender	−0.32	0.05	−6.30***	(−0.42, −0.22)
Group size	0.01	0.01	0.97	(−0.01, 0.02)
Grade	−0.07	0.06	−1.16	(−0.18, 0.05)
Leader's academic achievement	−0.11	0.05	−2.32*	(−0.19, −0.02)
Nonleader's academic achievement	−0.04	0.05	−0.91	(−0.14, 0.05)
Peer victimization				
Individual‐level				
Stability	0.68	0.06	11.74***	(0.56, 0.79)
Academic achievement	−0.08	0.03	−2.52*	(−0.15, −0.02)
Leadership status	−0.06	0.04	−1.61	(−0.13, 0.01)
Group‐level				
Gender	−0.16	0.05	−3.31**	(−0.26, −0.07)
Group size	0.00	0.01	−0.19	(−0.01, 0.01)
Grade	−0.01	0.06	−0.23	(−0.13, 0.10)
Leader's academic achievement	−0.06	0.04	−1.46	(−0.14, 0.02)
Nonleader's academic achievement	−0.18	0.06	−3.01**	(−0.29, −0.06)
Internalizing problems				
Individual‐level				
Stability	0.30	0.04	6.97***	(0.22, 0.39)
Academic achievement	−0.03	0.03	−1.01	(−0.10, 0.03)
Leadership status	−0.05	0.05	−1.14	(−0.14, 0.04)
Group‐level				
Gender	−0.02	0.04	−0.36	(−0.10, 0.07)
Group size	−0.01	0.01	−1.34	(−0.02, 0.00)
Grade	0.08	0.05	1.65	(−0.02, 0.18)
Leader's academic achievement	0.04	0.03	1.03	(−0.03, 0.10)
Nonleader's academic achievement	−0.13	0.04	−3.30**	(−0.21, −0.05)
Depression				
Individual‐level				
Stability	0.54	0.04	15.01***	(0.47, 0.61)
Academic achievement	−0.01	0.01	−1.19	(−0.03, 0.01)
Leadership status	−0.04	0.02	−1.98*	(−0.08, −0.01)
Group‐level				
Gender	0.04	0.02	1.94	(0.00, 0.08)
Group size	−0.01	0.00	−1.96	(−0.01, 0.00)
Grade	0.05	0.02	2.58*	(0.01, 0.10)
Leader's academic achievement	0.00	0.02	−0.24	(−0.04, 0.03)
Nonleader's academic achievement	−0.06	0.02	−2.58*	(−0.11, −0.01)

*Note*: Leadership status was coded as 0 = nonleader group member and 1 = peer group leader; gender was coded as 0 = predominantly male and 1 = predominantly female, grade was coded as 0 = Grade 7 and 1 = Grade 8.

* *p* < .05.

** *p* < .01.

*** *p* < .001.

To assess whether the focal associations varied by gender or grade, we added the following two‐way interaction terms to each multilevel model: (a) leadership status × gender, (b) leaders' academic achievement × gender, (c) nonleaders' academic achievement × gender, (d) leadership status × grade, (e) leaders' academic achievement × grade, and (f) nonleaders' academic achievement × grade. The interactions were not significant, indicating that the effects of peer group variables on the adjustment outcomes were consistent for boys and girls and for students in Grades 7 and 8.

## DISCUSSION

Peer groups have long been recognized as a crucial context for adolescent development in social and psychological domains (Rubin et al., 2015). It has been suggested that, relative to nonleader members, group leaders exert disproportionately greater influence due to their centrality and prestige on individual behaviors and development (Field et al., 2024). However, no research has been conducted to distinguish group leaders' contributions from those of nonleader members. The current study revealed that the academic achievement of both leaders and nonleader members predicted individual improved academic performance, social competence, and school competence over time. Moreover, leaders' academic achievement negatively predicted later aggression and externalizing behaviors, whereas nonleader members' achievement negatively predicted later peer victimization and internalizing symptoms. These findings indicated similar as well as different associations of group leaders' and nonleaders' academic achievement with adolescents' adjustment.

### Peer group leadership status and adjustment

The results first showed that peer group leaders displayed higher levels of academic achievement, social competence, school competence, and aggression and fewer internalizing problems at Time 1 than nonleader members. The higher levels of competence and aggression align with bi‐strategic Resource Control Theory (Hartl et al., 2020) and the prestige‐dominance model (Cheng et al., 2013), suggesting that effective leaders utilize both competence‐based assets and coercive strategies to consolidate their status. The fewer internalizing problems of group leaders suggest that these problems may undermine one's ability to function in the leadership role, such as active participation in group interactions and activities and providing support to others, which are essential for accruing and maintaining prestige and status in the group (Schwartz‐Mette et al., 2020).

Moreover, after accounting for baseline behaviors, leadership status positively predicted subsequent social and school competence and negatively predicted later depression. The results suggest that the experience of leadership primarily enhances positive social and psychological development. It is possible that leadership provides adolescents with opportunities to practice and enhance their social skills, such as interpersonal negotiation, decision‐making, and conflict‐resolution skills (King et al., 2009). Furthermore, leadership positions may bring about greater access to social and emotional resources, such as peer support and instrumental assistance, which likely foster development of social competence and psychological well‐being (Feinberg, 2024). The results indicated that leadership status did not significantly predict changes in academic achievement and aggression. Thus, the experience of leadership may not further increase the initial differences between group leaders and nonleaders on academic achievement and aggressive behavior. The results seem to suggest that although leaders may use academic achievement and aggressive behaviors during the process of leadership emergence, they may consider it unnecessary to continue to use them after their leadership status is secured (Hartl et al., 2020). Of course, this issue needs to be further investigated in the future.

### Contributions of peer group leaders' and nonleader members' academic achievement

The main purpose of this study was to extend previous research on peer group academic influence (e.g., Kindermann, 2007; Liu et al., 2023) by disentangling group leaders' effects from nonleaders' effects on adolescent adjustment. The independent predictive relations of both leaders' and nonleaders' academic achievement with subsequent academic, social, and school competence indicate dual mechanisms of peer influence. The status of leaders likely makes their behaviors particularly influential within the group (Field et al., 2024). Leaders' attitudes toward academic work, such as diligence, and associated achievement may establish expectations for others in the group. Moreover, high‐achieving leaders may organize collaborative learning activities, disseminate resources for academic performance, and explicitly endorse effort and persistence (Sabato & Kogut, 2021). These behaviors may help group members improve their learning skills and academic achievement. At the same time, the activities organized by group leaders may provide opportunities for group members to engage in constructive interactions for academically oriented goals, which in turn may help them improve their social skills and competence (Van Ryzin & Roseth, 2021). On the contrary, nonleader group members may influence each other through frequent interactions and mutual support in a relatively equalitarian manner. Interactions among nonleader members in academic activities, such as reciprocal assistance and collaboration on homework, may facilitate the formation of group norms on self‐control and cooperative behaviors (Chen et al., 2023; Low & Van Ryzin, 2024). According to Marsh et al. (2008), adolescents often assess their academic competence through comparisons upward with more capable peers, such as group leaders, as well as laterally with the “generalized other” or classmates. In this sense, the academic climate shaped by nonleader members helps establish group norms that reinforce engagement with shared practices. Participation in the group activities may promote effective communication, collaborative problem‐solving, and joint goal setting, which help develop social and school competence. Furthermore, high‐achieving nonleaders are likely to help peers alongside leaders, thereby broadening the group's collective access to academic and social resources and associating with positive developmental outcomes across domains (Chen et al., 2023; Ellemers, 2012; Liu et al., 2023).

Interestingly, our results showed distinct patterns of relations of group leaders' academic achievement and nonleader members' academic achievement with adolescents' externalizing and internalizing problems. Leaders' academic performance significantly and negatively predicted externalizing maladjustments likely through their influence to discourage aggressive, under‐controlled, and disruptive behaviors that may impede learning activities (Metsäpelto et al., 2015). Moreover, high‐achieving leaders might reduce the occurrence of unstructured group interactions as a context for aggression and externalizing problems through directing and organizing structured and academically focused activities (Gremmen et al., 2019; Xia & Ma, 2023).

The results indicated that the academic achievement of nonleader members predicted decline in peer victimization, internalizing symptoms, and depression. This finding suggests the critical role of social and emotional support provided by nonleader peers in adolescents' socioemotional adjustment. Frequent interactions with academically competent peers, such as working on and checking homework together and sharing emotions involved in academic work, might buffer against stress and distress and reduce victimization via fostering a sense of group cohesion and feelings of security (Chen et al., 2023; Rueger et al., 2016). By contrast, high‐achieving group leaders seem to be less effective in adolescents' internalizing problems and victimization, perhaps because their relatively more hierarchical interactions with group members weaken their ability to foster emotional closeness and security and group belonging among peer group members (Müller‐Kalthoff et al., 2017).

No significant gender or grade differences were found in this study, suggesting peer group leadership may function similarly for boys' and girls' groups and for groups in Grades 7 and 8. Future research should further examine potential developmental and gender variations in peer group functioning. For example, the literature suggests that boys' peer groups are generally more hierarchical and competitive, and girls' peer groups are more inclined to prioritize egalitarianism and interpersonal relationships (Rose & Rudolph, 2006), it will be important to continue to explore how gender plays a role in affecting the contributions of group leaders and members to adolescent development.

### Limitations and future directions

Several limitations of the present study should be acknowledged. First, the study assessed the predictive effects of peer group leaders' and nonleader members' academic achievement without tapping into the mechanisms or processes. Future research may incorporate direct measurement of these processes using behavioral observations, network analysis of support flows, qualitative interviews, and other methods to capture the social‐interactional and social‐cognitive processes, such as modeling, reinforcement, and collective goal setting, by which peer group leaders and nonleaders exert their influence.

Second, this study focused on peer groups in early adolescence. One should be careful in generalizing the findings to other developmental periods, such as middle childhood or late adolescence when peer groups may function differently (Laursen & Faur, 2022).

Third, this study was conducted in a Chinese cultural context, in which academic achievement is highly valued (Chen et al., 2023). It will be interesting to examine peer group leadership in other societies. Relatedly, this study was conducted in a rural region. In such context, most students may be from families with limited educational and social resources and thus peers may serve as particularly influential sources of academic and social support (Liu et al., 2018), potentially strengthening the roles of peer group leaders and members in adolescents' adjustment. Thus, generalization of the findings to other contexts should be made with caution, and it will be important to examine the associations between academic achievement of peer group leaders and nonleader members and adolescent adjustment in urban regions.

Fourth, the present study is correlational in nature. The terms “effects” and “contributions” were used in a statistical sense when describing the results. Thus, one needs to be careful in interpreting the results in terms of causality.

Fifth, because a primary purpose of the study was to examine the contributions of group leaders' academic achievement to group members' adjustment, the analyses were conducted with peer groups with clearly defined leadership structures. As a result, a substantial proportion of the original sample (the isolates and members of groups with only two members and groups without identifiable leaders) was not included in the formal analyses. This exclusion criterion may have important implications for interpretation of the results. By focusing on peer groups with identifiable leaders, the results may not generalize to peer groups that lack clear leadership structures. The findings of the present study should be understood mainly for socially connected adolescents embedded in peer groups with recognizable leadership. Future research should explore peer socialization in different types of group contexts from a broader perspective.

Sixth, we focused on the contributions of group leaders' and nonleaders' academic achievement to later individuals' adjustment, with their own initial academic achievement and adjustment controlled, in this study, which is consistent with the argument that once formed, peer groups play a significant role in shaping children's and adolescents' development (Rubin et al., 2015). Nevertheless, an important issue in understanding peer groups is how they are formed. Researchers should examine factors that contribute to the formation of peer groups and the emergence of leadership in Chinese adolescents. For example, it will be interesting to collect data on adolescents' attributes when they start middle school, assess peer group affiliation and leadership status in a follow‐up study, and examine longitudinal associations between adolescents' attributes and group affiliation and leadership status.

Finally, the current study focused on academic achievement of group leaders and nonleader members. Researchers should study group leaders' and members' other attributes, such as aggression and emotional competence, which will provide a more comprehensive understanding of peer influence. In addition, peer groups may also differ in their primary purposes (e.g., interest‐based, friendship‐based, academic support‐based groups), which may affect the salience of leaders' and members' characteristics. For example, whereas academic achievement may be particularly relevant in academically oriented groups, dyadic social skills may be more central in friendship‐based groups. It will be interesting to incorporate measures of group purpose in future research to achieve a more comprehensive understanding of peer groups as a developmental context.

Despite these limitations, the results of the present study help us understand how peer group leaders' and nonleader members' academic achievement shape adolescent development in academic, social, and psychological domains. The results also have practical implications for educators and professionals working with adolescents. For example, our results indicated that the academic achievement of both group leaders and nonleader members positively predicted adolescents' social competence and academic achievement. At the same time, peer group leaders' academic achievement negatively predicted adolescents' aggression and externalizing problems, whereas nonleader members' academic achievement negatively predicted adolescents' victimization and internalizing problems. These results suggest that group‐based approaches that engage group leaders and group members in education and intervention programs may be useful for supporting adolescents' adjustment. Promoting academic activities in peer groups in general is likely to be conducive to adolescents' development of academic as well as social competence. Moreover, it may be a particularly effective strategy to encourage group leaders to promote academically oriented group goals and organize group activities on academic tasks to help adolescents reduce aggressive behaviors and externalizing problems. Moreover, teachers and professionals should also create an environment and provide opportunities to facilitate cooperative academic engagement among peer group members to reduce victimization and internalizing problems.

## AUTHOR CONTRIBUTIONS

**Dan Li:** Data curation; project administration; supervision; software; resources. **Junsheng Liu:** Supervision; project administration; resources. **Jiaxi Zhou:** Conceptualization; methodology; writing – review and editing; writing – original draft; formal analysis; visualization.

## FUNDING INFORMATION

This research received no funding.

## CONFLICT OF INTEREST STATEMENT

The authors report no conflict of interest.

## ETHICS STATEMENT

This research complied with the APA's ethical standards. The ethical approval was obtained from the Institutional Review Board of Shanghai Normal University.

## PATIENT CONSENT STATEMENT

Parent/guardian consent was obtained.

## Supporting information


**Data S1.** Supporting Information.

## Data Availability

The datasets generated and/or analyzed during the current study are not publicly available but are available from the corresponding author upon reasonable request.
