# Supplementary material for: Academic achievement of peer group leaders and members: Contributions to adolescents' social, school, and psychological adjustment
Source: J Res Adolesc. 2026 Jan 14;36(1):e70143. doi: 10.1111/jora.70143 (PMC12801095; doi:10.1111/jora.70143)
Supplement: Supplementary file 1 — Data S1. Supporting Information. [file JORA-36-0-s001.docx]

Academic Achievement of Peer Group Leaders and Members: Contributions to Adolescents’ Social, School, and Psychological Adjustment

**Supplementary Materials**

The multilevel model examining the effects of individual-level and peer group-level variables at Time 1 on individual-level adjustment at Time 2 is as follows:

Level 1 (within-group level):
T2Y_ij_=β_0j_+β_1j_T1Y_ij_+β_2j_AcademicAchievement_ij_+β_3j_GroupLeadershipStatus_ij_+ϵ_ij_
Level 2 (between-group level):
β_0j_=γ_00_+γ_01_GroupGender_j_+γ_02_GroupSize_j_+γ_03_Grade_j_+γ_04_GroupLeaderAcademicAchievement_j_+γ_05_NonLeaderAcademicAchievement_j_+μ_0j_
β_1j_= γ_10_+μ_1j_
β_2j_= γ_20_+μ_2j_
β_3j_= γ_30_+μ_3j_

The term T2Y_ij_ represents the adjustment outcome variable at Time 2 for adolescent i in peer group j. T1Y_ij_ indicates the corresponding adjustment variable at Time 1 for adolescent i in peer group j. AcademicAchievementij indicates the academic achievement at Time 1 for adolescent i in peer group j. GroupLeadershipStatus_ij_ represents Time 1 peer group leadership status for adolescent i in peer group j. GroupGender_j_, GroupSize_j_, and Grade_j_ denote the gender composition, size of peer group, and grade for group j at Time 1. GroupLeaderAcademicAchievement_j_ and NonLeaderAcademicAchievement_j_ represent Time 1 group leaders’ academic achievement and the average academic achievement of nonleader group members, respectively, for peer group j. Random effects (μ_0j_, μ_1j j_, μ_2j j_, and μ_3j_) capture between-group variation in the intercept and slopes, allowing the predictive effects of prior adjustment, academic achievement, and leadership status to vary across groups.

Table S1.

*Multilevel Estimates of Time 1 Group Differences Between Peer Group Leaders and Nonleaders*

| T1 Outcome Variable | *B* | *SE* | *t value* | *95% CI* |
| --- | --- | --- | --- | --- |
| Academic achievement | 0.43 | 0.05 | 8.25*** | (0.33, 0.53) |
| Social competence | 1.15 | 0.10 | 11.53*** | (0.96, 1.35) |
| School competence | 0.28 | 0.05 | 6.12*** | (0.19, 0.37) |
| Aggression | 0.24 | 0.06 | 4.19*** | (0.13, 0.35) |
| Externalizing problems | -0.08 | 0.04 | -1.76 | (-0.16, 0.01) |
| Peer victimization | -0.06 | 0.06 | -1.10 | (-0.17, 0.05) |
| Internalizing problems | -0.12 | 0.04 | -3.05** | (-0.20, -0.04) |
| Depression | -0.03 | 0.02 | -1.70 | (-0.07, 0.01) |

*Note*. Positive *B* values indicate that peer group leaders had higher scores than nonleader group members. Estimates are based on multilevel models with leadership status as the predictor, controlling for peer group gender composition, group size, and grade level.

***p* < .01. ****p* < .001.

*Figure S1*. Conceptual Model of the Study.
